# DAEF-YOLO Model for Individual and Behavior Recognition of Sanhua Geese in Precision Farming Applications

**DOI:** 10.3390/ani15203058

**Published:** 2025-10-21

**Authors:** Tianyuan Sun, Shujuan Zhang, Rui Ren, Jun Li, Yimin Xia

**Affiliations:** 1 College of Agricultural Engineering, Shanxi Agricultural University, Taigu District, Jinzhong 030801, China; z20223024@stu.sxau.edu.cn (T.S.); b20221003@stu.sxau.edu.cn (R.R.); lijun@sxau.edu.cn (J.L.); 2 Dryland Farm Machinery Key Technology and Equipment Key Laboratory of Shanxi Province, Taigu District, Jinzhong 030801, China; 3 College of Software Engineering, Shanxi Agricultural University, Taigu District, Jinzhong 030801, China; xiayimin@sxau.edu.cn

**Keywords:** deep learning, poultry, precision farming, object detection, feature extraction

## Abstract

**Simple Summary:**

Individual and behavior recognition are essential techniques in modern livestock and poultry farming, supporting precision agriculture and improving production efficiency. This study develops an improved YOLOv8-based model, named DAEF-YOLO (DualConv-augmented C2f, ADown down-sampling, Efficient Channel Attention integrated into SPPF, and FocalerIoU regression loss), to address the challenges of multi-task recognition and real-time monitoring in Sanhua goose farming. The model can simultaneously recognize individual geese and multiple behaviors while maintaining a balanced trade-off between accuracy and efficiency. It achieved individual recognition performance comparable to single-task detectors, with behavior recognition precision of 94.65% and mAP@0.5 of 96.10%. These results demonstrate that DAEF-YOLO can effectively support automated monitoring and intelligent management for precision goose farming.

**Abstract:**

The rapid expansion of the goose farming industry creates a growing need for real-time flock counting and individual-level behavior monitoring. To meet this challenge, this study proposes an improved YOLOv8-based model, termed DAEF-YOLO (DualConv-augmented C2f, ADown down-sampling, Efficient Channel Attention integrated into SPPF, and FocalerIoU regression loss), designed for simultaneous recognition of Sanhua goose individuals and their diverse behaviors. The model incorporates three targeted architectural improvements: (1) a C2f-Dual module that enhances multi-scale feature extraction and fusion, (2) ECA embedded in the SPPF module to refine channel interaction with minimal parameter cost and (3) an ADown down-sampling module that preserves cross-channel information continuity while reducing information loss. Additionally, the adoption of the FocalerIoU loss function enhances bounding-box regression accuracy in complex detection scenarios. Experimental results demonstrate that DAEF-YOLO surpasses YOLOv5s, YOLOv7-Tiny, YOLOv7, YOLOv9s, and YOLOv10s in both accuracy and computational efficiency. Compared with YOLOv8s, DAEF-YOLO achieved a 4.56% increase in precision, 6.37% in recall, 5.50% in F1-score, and 4.59% in mAP@0.5, reaching 94.65%, 92.17%, 93.39%, and 96.10%, respectively. A generalizable classification strategy is further introduced by adding a complementary “Other” category to include behaviors beyond predefined classes. This approach ensures complete recognition coverage and demonstrates strong transferability for multi-task detection across species and environments. Ablation studies indicated that mAP@0.5 remained consistent (~96.1%), while mAP@0.5:0.95 improved in the absence of the “Other” class (75.68% vs. 69.82%). Despite this trade-off, incorporating the “Other” category ensures annotation completeness and more robust multi-task behavior recognition under real-world variability.

## 1. Introduction

In recent years, the rapid advancement of intelligent agriculture and the growing consumer demand for high-quality food have brought increasing attention to intelligent monitoring in poultry farming, which is crucial for enhancing production efficiency and ensuring product quality [1,2]. Geese, as valuable waterfowl species possessing both high nutritional value and substantial economic potential, occupy an important position in the poultry industry [3]. Among them, individual and behavior recognition play a critical role in farm asset evaluation, offering essential data for precision feeding, improving production performance, and supporting overall farm management efficiency [4,5,6].

Traditional approaches for recognizing individual animals and their behaviors have traditionally relied on manual observation and statistical sampling. Such methods are time-consuming, labor-intensive, and prone to human bias [6,7], making them inadequate for the intelligent, efficient, and large-scale management required in modern farming. To overcome these limitations, researchers have increasingly adopted computer vision-based techniques to achieve automated animal monitoring [8]. Existing recognition techniques can generally be categorized into two types: contact-based sensor monitoring and non-contact visual monitoring [3,9]. Sensor-based methods require installing devices on animals to collect information such as movement, temperature, and physiological signals. However, attaching or implanting sensors can introduce mechanical noise or vibration during data collection and may trigger stress responses, thereby compromising animal welfare.

In contrast, vision-based non-contact monitoring leverages cameras and deep learning models to analyze images and videos, enabling real-time and accurate recognition without disturbing the animals [10]. For instance, Gao et al. combined CNN and GRU to recognize aggressive behaviors in group-housed pigs [11], while Fang et al. employed the CenterNet model for pig recognition and behavioral analysis [12]. These approaches have been effectively applied to a range of tasks, including livestock and poultry individual recognition and counting [13,14,15,16], body condition detection [9,17,18,19], behavior recognition [7,20,21,22,23], and welfare assessment [24,25]. For example, Huang et al. integrated DenseNet into the Single Shot MultiBox Detector (SSD) and applied a particle–Kalman filter fusion algorithm, achieving accurate dairy cow counting [17]. Fang et al. evaluated broiler chicken posture classification using skeleton recognition technology, achieving recognition accuracies of 75.11% (standing), 51.35% (walking), 62.70% (running), 93.61% (eating), 96.23% (resting), and 92.58% (pecking feathers) [12]. Similarly, Ye et al. applied YOLO and MRM to detect stupor levels in chickens, achieving an accuracy of 96.77% [26].

However, despite the success of these methods in other species, research focusing on geese remains relatively limited [4,5,6,27]. Giannone et al. [28] recently demonstrated the feasibility of YOLO-based [29,30,31] methods in precision farming by applying an improved YOLOv8n model for dairy cow identification and feeding behavior monitoring. Nevertheless, goose-related studies still face several limitations, such as restricted deployment, single-task orientation, and idealized experimental settings. These issues underscore the urgent need for solutions capable of real-time validation, multi-task processing, and robust generalization under complex environmental conditions.

Advances in general-purpose object detection and behavior recognition also provide valuable insights for livestock applications. For example, an improved SSD-like network for indoor object detection demonstrated efficient multi-scale feature extraction and lightweight optimization [32]. Similarly, a 3D Dense Connections framework for abnormal behavior recognition emphasized the importance of spatiotemporal modeling in complex activity analysis [33]. Other studies, including GRU-based gesture recognition on skeleton dynamic graphs [34] and skeleton-aware driver behavior frameworks [35], highlighted the value of integrating temporal and structural cues for fine-grained recognition. However, such models depend heavily on skeleton or sequential data, which are difficult to obtain in large-scale poultry farming environments. In contrast, the proposed DAEF-YOLO directly processes raw RGB images, offering a unified and deployable solution for simultaneous individual and behavior recognition in geese. It effectively addresses key challenges such as real-time validation, multi-task processing, and generalization in complex farming conditions.

To tackle these challenges, this study introduces a YOLOv8-based model designed to perform simultaneous individual and behavior recognition of *Sanhua geese*, achieving an optimal balance between high accuracy and deployment efficiency in real-world conditions.

The main contributions are as follows:

(1) Dedicated goose dataset: We constructed a high-quality dataset comprising multi-scale images of Sanhua goose individuals and behaviors captured under realistic and complex farming conditions. The dataset includes ten behavior categories—Drinking, Feather Preening, Feeding, Floating, Grooming, Pecking, Resting, Standing, Wing Stretching, and Other—serving as a robust foundation for multi-task model training and extending the behavioral taxonomy of geese in current research.
(2) Multi-task recognition strategy: We propose a generalizable classification framework that introduces an “Other” category as a complementary class within a clearly defined multi-behavior system. This ensures that ambiguous or undefined behaviors are properly categorized, allowing for complete and simultaneous recognition of all individuals and behaviors. The strategy maintains individual recognition performance comparable to single-class detection while providing strong transferability and scalability for other species and scenarios, thereby supporting intelligent livestock management.
(3) Improved DAEF-YOLO architecture: Based on the YOLOv8s backbone, we implement targeted structural optimizations for multi-task scenarios. The DualConv-enhanced C2f module improves multi-scale feature extraction [36]; ECA within the SPPF module enhances channel interaction with minimal parameter cost [37]; the ADown module preserves information during downsampling [30], and the FocalerIoU loss improves bounding-box regression accuracy under complex backgrounds [38]. This integrated architecture achieves significant accuracy gains while retaining lightweight and real-time performance characteristics.

## 2. Materials and Methods

### 2.1. Image Acquisition and Dataset Construction

**Figure 1:**
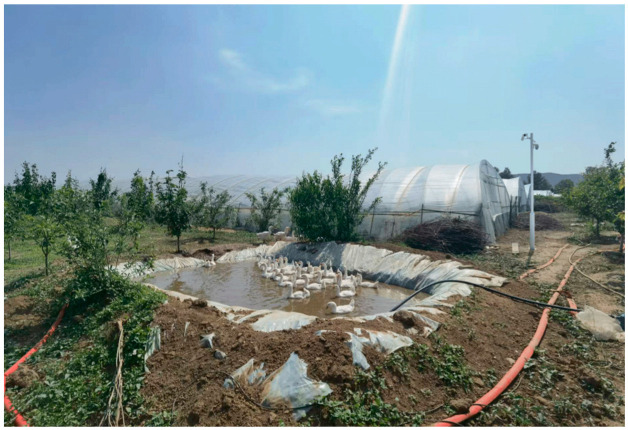
Data collection environment.

This study was conducted at a commercial goose farm in Shanxi Province, China. A total of 150 healthy *Sanhua geese*, each 45 days old, were raised under a semi-free-range management system throughout the experimental period. During daytime (08:00–20:00), the geese freely moved around a designated pond, returning to the goose house at night. Feeding was scheduled twice per day, at 09:00–09:20 and 18:00–18:20. The study area is shown in Figure 1.

**Figure 2:**
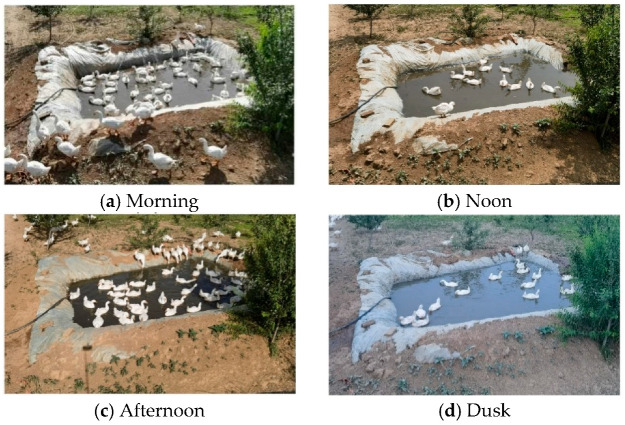
Example of shooting frame.

Video data were continuously collected from May 10 to June 1, 2024, using a Hikvision camera (DS-2CD3T86FWDV3-LS; Hangzhou Hikvision Digital Technology Co., Ltd., Hangzhou, China) with 3840 × 2160 resolution, 25 FPS, and a 70° downward angle at a height of 3.7 m. Video frames were extracted at a 1:100 interval throughout the observation period, generating 854 high-quality representative images (Figure 2). Subsequently, data augmentation techniques—including brightness adjustment, rotation, mirroring, and noise addition—were applied, yielding a final dataset of 1720 annotated images.

To prevent data leakage, the dataset was split at the recording-session level. All frames from a single continuous recording session were assigned to the same subset (training, validation, or testing), ensuring that temporally adjacent frames did not overlap across subsets. This temporal separation minimized near-duplicate leakage and improved the reliability of model evaluation.

Ten categories were defined: Drinking, Feather Preening, Feeding, Floating, Grooming, Pecking, Resting, Standing, Wing Stretching, and Other. The “Other” category encompassed postures that did not clearly belong to the nine predefined behaviors, thereby ensuring comprehensive labeling of all visible individuals. Detailed behavioral definitions and representative examples are provided in Table 1. Annotation was conducted with the LabelImg tool according to Pascal VOC standards, and individuals with body occlusion greater than 75% were excluded [39].

The classification approach reflects a generalizable multi-task detection strategy. Within a clearly defined multi-class behavioral system, an additional complementary class was introduced to capture instances outside the predefined categories. This design ensured annotation completeness, maximized data utilization, and mitigated the effect of rare-class imbalance on overall model performance. Consequently, the framework achieved stable multi-task detection while retaining fine-grained recognition capability. This strategy also demonstrates strong transferability and scalability, allowing its application to other species, farming conditions, and domains.

**Figure 3:**
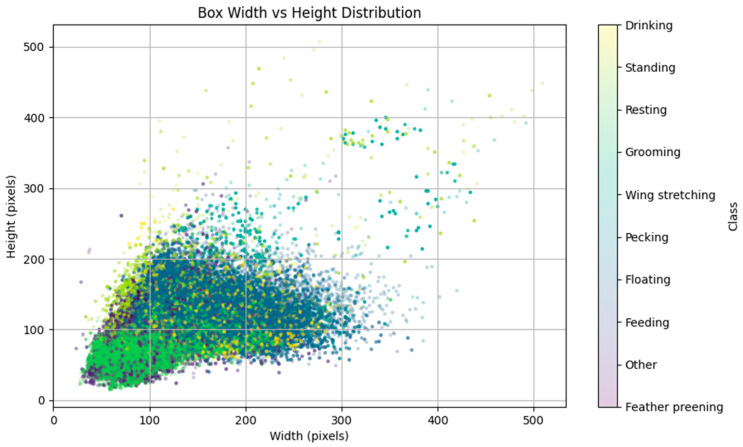
Distribution of bounding box dimensions across ten classes.

The dataset comprised 109,888 labeled instances (Table 2). Among them, Floating (38.26%) and Resting (37.81%) were the most frequent behaviors. A scatter plot of bounding box width and height distributions (Figure 3) revealed class-specific patterns, providing insights into scale-aware model optimization and anchor-free detection design. On average, each image contained ~63.9 annotated instances, with bounding boxes covering only ~0.177% of the total image area.

Individuals with greater than 75% body occlusion were excluded. For partially occluded geese (<75%), bounding boxes were drawn tightly around the visible region, ensuring inclusion of the head–neck or trunk when visible. All annotations were performed by the first author following consistent guidelines, ensuring dataset-wide labeling uniformity.

As shown in Table 2, the dataset exhibited a clear imbalance, with Floating (38.26%) and Resting (37.81%) together accounting for more than 75% of all annotations, while minority behaviors such as Grooming (1.03%) and Wing stretching (0.83%) were under-represented. Such imbalance may bias the model toward majority classes and hinder the recognition of rare behaviors. In this study, the classification branch used the default BCE loss without focal modulation (γ = 0) or class re-weighting, while the regression branch employed the proposed FocalerIoU loss. To enhance robustness, a uniform set of augmentations (rotation, mirroring, brightness adjustment, cutout, etc.) was applied to all images and instances. The “Other” category was introduced mainly to ensure that all individuals could be recognized alongside multiple behaviors, rather than to address imbalance. Nevertheless, imbalance remains a potential limitation, and future work will investigate explicit class re-weighting, focal classification loss, and oversampling strategies to improve the performance of rare classes.

### 2.2. Data Augmentation

Online stochastic data augmentations were applied to each sample, ensuring at least one transformation per image. Specifically, the following operations were implemented: rotation (±5° with 0.70–0.80 scaling), translation within valid margins, brightness scaling (α = 0.35–1.00), Gaussian noise addition, cutout (one 50-pixel square; IoU ≤ 0.50 with any bounding box), and vertical or horizontal flipping. Mosaic and mix-up augmentations were intentionally omitted to avoid distributional bias. All randomness was controlled by fixed random seeds at the beginning of each run. The complete parameter ranges, trigger probabilities, and implementation details are summarized in Table S1.

### 2.3. Ablation Protocol on the “Other” Complement Class

To evaluate the effect of introducing the “Other” category as a complementary behavior class, a controlled ablation experiment was conducted.

Two configurations were trained under identical pipelines:

(i) a 10-class taxonomy including “Other”, and

(ii) a 9-class taxonomy excluding it.

Apart from the taxonomy, all other training parameters—including data partitioning (training/validation/testing), input resolution (1280 × 1280), augmentation strategies (rotation, mirroring, brightness adjustment, cutout, noise addition), optimizer, learning-rate schedule (cosine annealing with warm-up), exponential moving average (EMA), non-maximum suppression (NMS) thresholds, total epochs, and COCO-pretrained initialization—were kept identical.

### 2.4. YOLOv8 Model and Performance Comparison

YOLOv8, released by Ultralytics in 2023, was adopted as the baseline model for this study [29]. The network architecture comprises a backbone (C2f modules and SPPF), a neck (feature pyramid and path-aggregation fusion), and a decoupled detection head. Among its five variants, YOLOv8s was chosen for its favorable balance between accuracy (mAP@0.5 = 91.51%) and real-time inference capability (FPS = 74.3), as summarized in Table 3.

### 2.5. Construction of the Proposed DAEF-YOLO Model

An enhanced object-detection framework, named DAEF-YOLO, was developed based on the YOLOv8 architecture to address multi-task recognition challenges in *Sanhua goose* detection under complex farming conditions.

The model integrates: (1) a lightweight DualConv-enhanced C2f module to improve local spatial feature extraction; (2) an SPPF module augmented with Efficient Channel Attention (ECA) to reinforce global context representation; and (3) a simplified ADown down-sampling structure that minimizes information loss in early layers while maintaining cross-channel continuity. Furthermore, a novel FocalerIoU loss function was introduced to emphasize difficult samples, thereby improving bounding-box regression and localization accuracy in overlapping instances.

**Figure 4:**
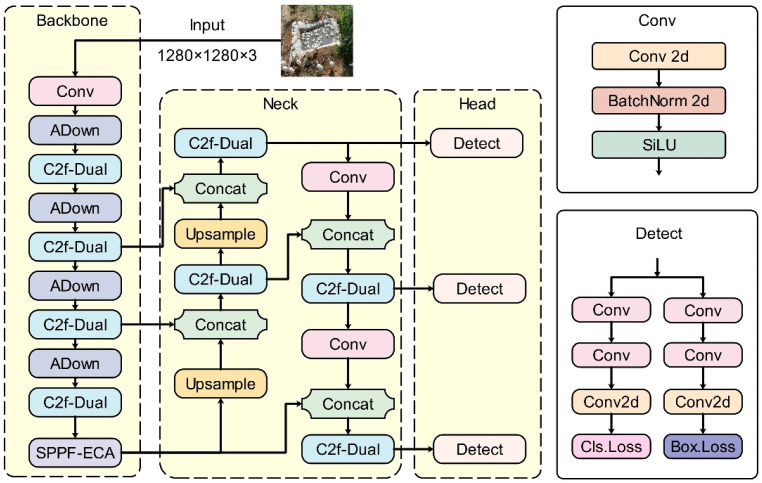
Structure of DAEF-YOLO model.

The overall network architecture of DAEF-YOLO is shown in Figure 4.

#### 2.5.1. C2f-Dual Module Based on DualConv

To enhance multi-scale feature extraction and spatial detail preservation while maintaining computational efficiency, the standard C2f block in YOLOv8 was redesigned into a dual-branch convolutional structure, termed C2f-Dual. Within this module, the original single convolution operation was replaced by a DualConv structure composed of parallel 3 × 3 and 1 × 1 convolutions. This configuration allows the network to learn coarse- and fine-grained representations simultaneously. The two branches are concatenated and fused with a residual shortcut, which improves feature diversity, maintains gradient flow, and reduces redundancy.

**Figure 5:**
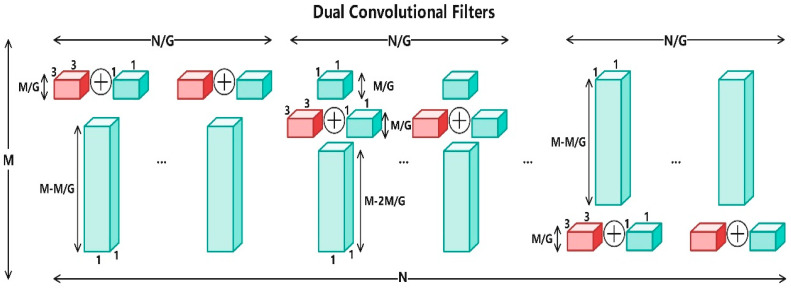
DualConvolution Architecture Visualization.

**Figure 6:**
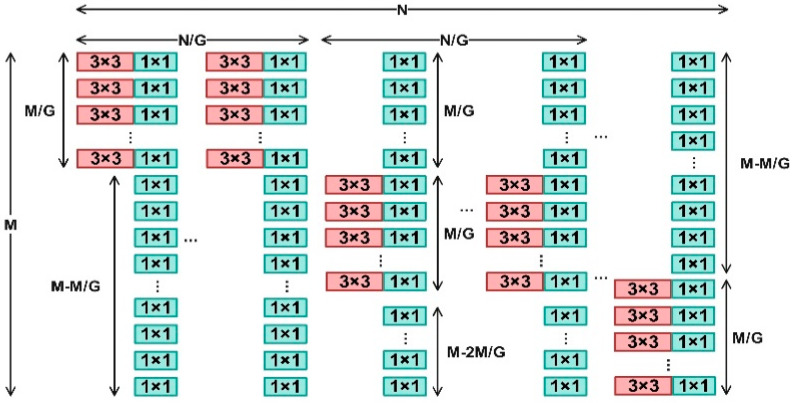
Parallel layout of 3 × 3 and 1 × 1 convolutional kernels in DualConv.

**Figure 7:**
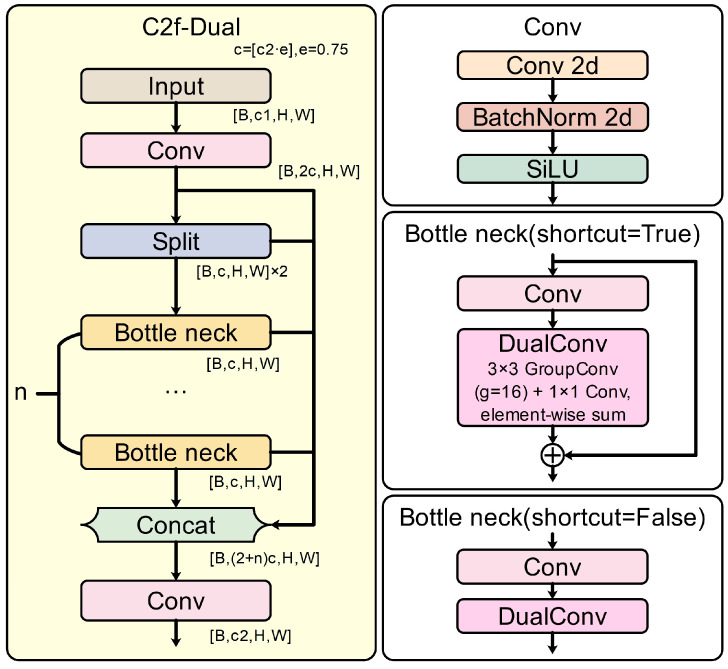
Block diagram of the proposed C2f-Dual module with explicit tensor shapes. The input [B, c1, H, W] is first expanded by a 1 × 1 convolution to [B, 2c, H, W], where c = ⌊c2·e⌋ and e = 0.75. The tensor is split into two [B, c, H, W] branches; one branch is concatenated directly while the other passes through n Bottleneck blocks, each embedding a DualConv operator (3 × 3 group convolution + 1 × 1 convolution, summed). The outputs are concatenated to [B, (2 + n)c, H, W] and compressed by a final 1 × 1 convolution to [B, c2, H, W].

As illustrated in Figure 5, Figure 6 and Figure 7, the dual-branch design facilitates efficient gradient propagation through residual fusion, and explicit tensor dimensions are provided in the schematic for reproducibility. Compared with the vanilla C2f block, C2f-Dual markedly improves the model’s capability to distinguish subtle or overlapping goose behaviors, especially under occlusion and in dense scenes. As shown in Table 4, integrating C2f-Dual at one or more positions slightly increases the model size and FLOPs but brings substantial accuracy improvements. This demonstrates a favorable balance between precision and efficiency, confirming that C2f-Dual is practical for deployment on resource-limited devices.

#### 2.5.2. Improved SPPF Module with ECA

The original Spatial Pyramid Pooling-Fast (SPPF) module effectively aggregates multi-scale context but does not provide channel-wise feature recalibration. To overcome this limitation, an Efficient Channel Attention (ECA) mechanism was embedded into the SPPF output branch, producing the enhanced SPPF-ECA module.

**Figure 8:**
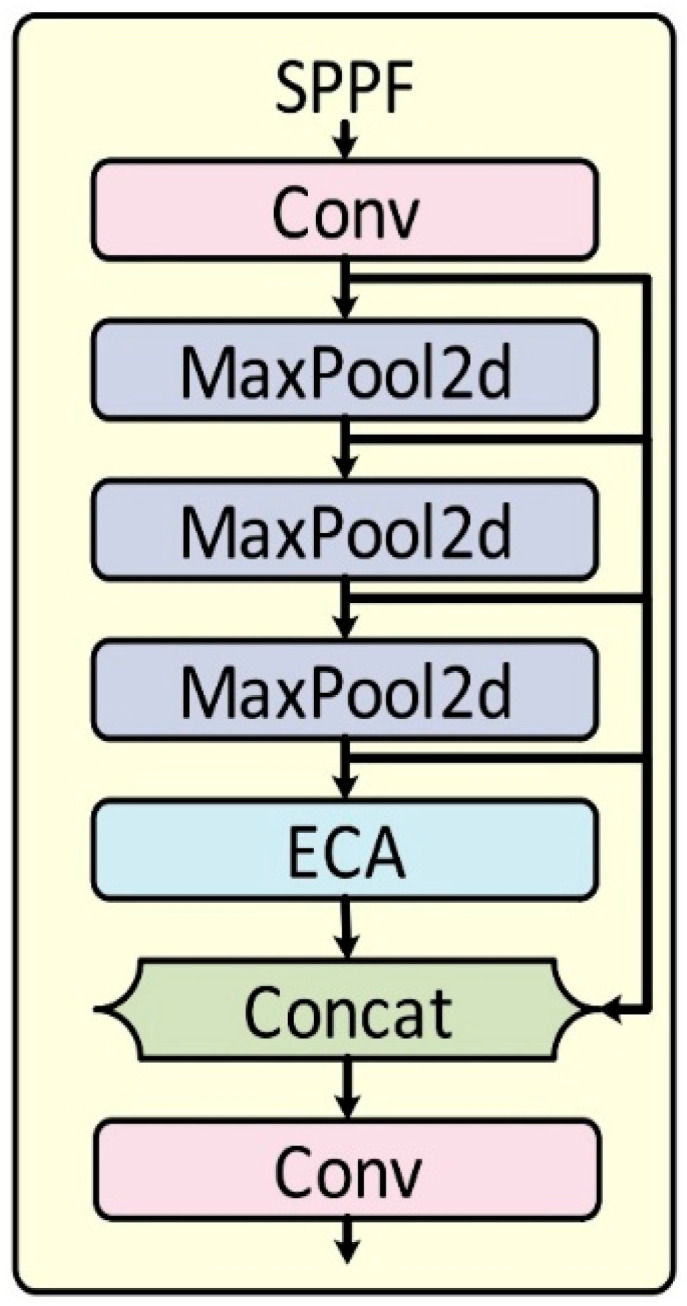
The structure of improved SPPF-ECA.

**Figure 9:**
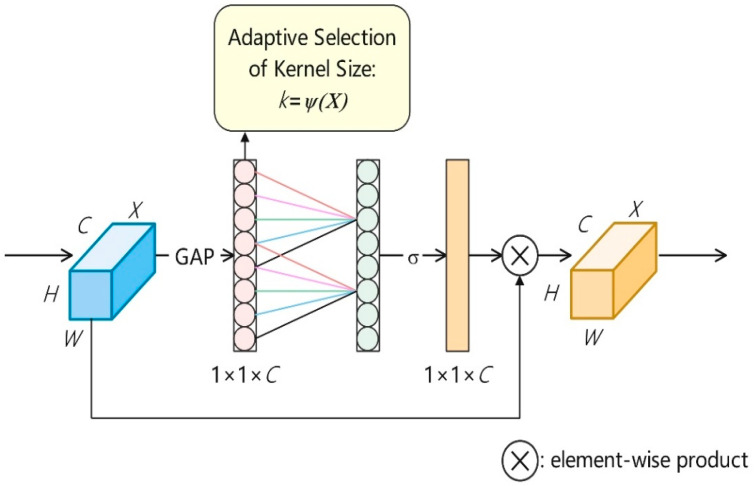
Diagram of ECA (efficient channel attention) module.

As illustrated in Figure 8, the standard SPPF structure was retained but augmented with the ECA block (Figure 9). The ECA mechanism adaptively re-weights channels through a lightweight 1-D convolution that models local cross-channel interactions. In this work, the kernel size was fixed at 3 and no reduction ratio was applied, maintaining high computational efficiency while strengthening attention to semantically important features. This enhancement enables the network to focus on more discriminative channels with negligible computational overhead.

#### 2.5.3. ADown Module for Downsampling

**Figure 10:**
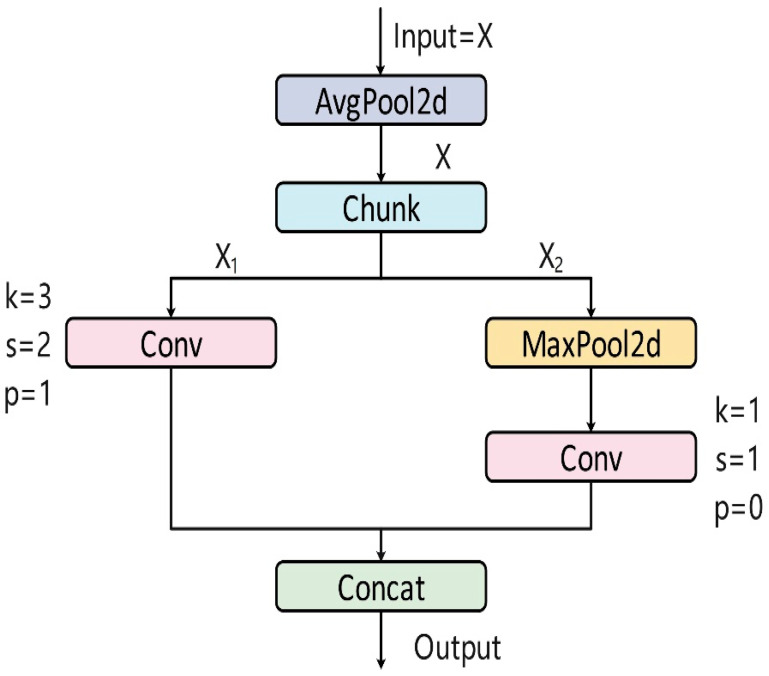
ADown structure principle.

To reduce the information loss typically caused by conventional down-sampling operations (e.g., max-pooling or high-stride convolution), an ADown module (Figure 10) was introduced. This module combines a 1 × 1 convolution for channel compression with a stride-2 depthwise convolution that better preserves edge and texture information.

In the improved backbone, ADown replaces standard down-sampling layers at four transition stages:

(i) after the initial Conv layer (64 → 128 channels, forming the P2/4 feature map);

(ii) after the first C2f-Dual block (128 → 256 channels, forming P3/8);

(iii) after the second C2f-Dual block (256 → 512 channels, forming P4/16); and

(iv) after the third C2f-Dual block (512 → 1024 channels, forming P5/32).

These explicit placements ensure cross-channel continuity and minimize information loss—particularly important for fine-grained detection tasks such as individual *Sanhua goose* identification.

#### 2.5.4. FocalerIoU Loss Function

Early object detection loss functions evolved from IoU [40], which measures overlap between predicted and ground truth boxes, to GIoU [41], DIoU [42], CIoU [43], and SIoU [44], each introducing improvements such as accounting for distance, aspect ratio, and orientation. While these methods enhance regression by leveraging geometric relationships, they generally neglect the imbalance between easy and hard samples, which can impair performance. To overcome this limitation, this study proposes the FocalerIoU loss, which dynamically adjusts loss values based on IoU, emphasizing harder regression samples and thereby improving detection performance across diverse tasks.

The piecewise IoU scaling term is defined with two thresholds d and u, which down-weight overly easy samples (IoU > u) and cap the effect of very hard samples (IoU < d). The final regression loss is written as:

(1) $$L_{FocalerIoU}=1-{\left({IoU}_{focaler}\right)}^{\gamma }$$

where ${IoU}_{focaler}$ is the scaled IoU term, and γ controls the focusing strength. In this study, parameters were set to d = 0.00, u = 0.60, and γ = 2.0. Following the common practice in focal-based losses [45], γ was set to 2.0, as preliminary experiments on our goose dataset confirmed that this value provided a stable trade-off between focusing strength and convergence. The loss fully replaces CIoU in the YOLOv8 baseline, without additional combination terms. This formulation directly follows the focal modulation principle, emphasizing hard samples and reducing gradients from easy ones, without introducing additional derivation complexity.

To evaluate the effectiveness of FocalerIoU, we compared it against several common IoU-based losses (CIoU, DIoU, SIoU, GIoU) under identical training settings. Results are summarized in Table 5.

As shown, FocalerIoU improves Precision from 90.09% to 91.54% (+1.45 percentage points), Recall from 85.80% to 87.13% (+1.33), F1 from 87.89% to 89.28% (+1.39), and mAP@0.5 from 91.51% to 93.26% (+1.75) compared with the CIoU baseline.

## 3. Results and Analysis

### 3.1. Experimental Platform

All model training and evaluation were performed in a high-performance computing environment to ensure the efficient processing of the DAEF-YOLO architecture. The experiments ran on Ubuntu 20.04.6, powered by an AMD EPYC 7542 32-core processor and two NVIDIA GeForce RTX 4090 GPUs (24 GB each). Implementation was carried out in Python 3.11 using the PyTorch 2.2.1 deep learning framework, with CUDA 12.1.1 for GPU acceleration. Models were trained for 100 epochs with a step-based learning rate schedule and a linear warm-up of 3 epochs. Neither Exponential Moving Average (EMA) nor early stopping was applied, and all training used full precision (FP32) without mixed precision acceleration (Table 6).

To ensure reproducibility and mitigate training stochasticity, all experiments were repeated three times using different random seeds. Results reported in the main text are presented as mean ± standard deviation (SD), or as mean values when the variation across runs was below 0.5%.

### 3.2. Evaluation Indicators

To comprehensively evaluate model performance, standard object detection metrics were adopted: Precision (P), Recall (R), F1-score, and mean Average Precision at an IoU threshold of 0.5 (mAP@0.5).

Precision (P): the proportion of correctly predicted positive samples among all predicted positives.

Recall (R): the proportion of actual positive samples correctly identified by the model.

F1-score: the harmonic mean of Precision and Recall, providing a balanced measure of performance.

mAP@0.5: the average precision across all categories at a fixed IoU threshold of 0.5, commonly used in detection tasks.

The metrics are formally defined as follows:

(2) $$P=\frac{T_{p}}{T_{p}+F_{p}}$$

(3) $$R=\frac{T_{p}}{T_{p}+F_{n}}$$

(4) $$F1=\frac{2\times P\times R}{P+R}$$

(5) $$\mathrm{mA}P=\frac{1}{N}\int_{0}^{1}P(R)dR$$

In addition to mAP@0.5, we also report mAP@0.5:0.95, which averages AP over IoU thresholds from 0.5 to 0.95 with a step of 0.05, providing a stricter and more comprehensive evaluation of detection performance.

In this study, True Positive (TP), False Positive (FP), False Negative (FN), and True Negative (TN) values were derived by comparing model predictions against ground-truth labels. The 10-category multi-task framework simultaneously assessed both individual and behavior recognition.

All metrics were computed under varying occlusion, illumination, and annotation conditions to ensure a robust quantitative evaluation.

### 3.3. Ablation Study on the Model’s Performance

A series of ablation experiments were conducted to quantify the contribution of each module within the DAEF-YOLO framework. The C2f-Dual, SPPF-ECA, FocalerIoU, and ADown components were progressively integrated into the YOLOv8s baseline to evaluate their individual and synergistic effects on detection accuracy, model size, computational complexity (FLOPs), and frame rate (FPS). Each configuration was tested independently and in combination, with experiments repeated three times on the validation set. Since the variation among runs remained below 0.5%, Table 7 reports only mean values for clarity.

The results reveal several key findings. Integrating the C2f-Dual module significantly enhanced feature extraction, improving both precision and F1-score. The SPPF-ECA module strengthened attention mechanisms and yielded noticeable gains in mAP@0.5 with minimal computation cost. The FocalerIoU loss improved bounding-box regression and boosted sensitivity to object overlap, while the ADown module—though increasing model complexity—preserved multi-scale feature information and led to additional accuracy gains. Among all configurations, Model H, which integrated all four proposed modules, achieved the highest overall performance with mAP@0.5 = 96.10%, precision = 94.65%, and F1 = 93.39%. These values match those in Table 7, as the final integrated model was further evaluated on an independent test set to ensure fair baseline comparison.

**Figure 11:**
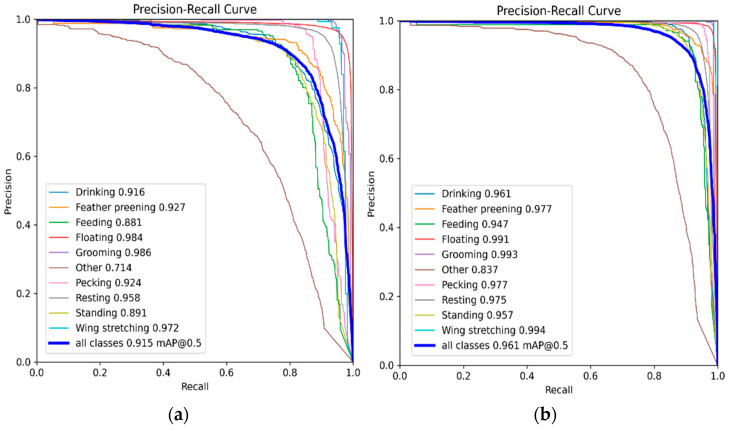
Per-class Precision-Recall (PR) curves for (**a**) the baseline YOLOv8s model and (**b**) the improved DAEF-YOLO model, covering ten *Sanhua goose* behavior categories and the overall mAP@0.5.

Figure 11 illustrates the per-class Precision–Recall (PR) curves for both the baseline YOLOv8s and the improved DAEF-YOLO models, evaluated on the test set. Across all ten behavioral categories, the proposed DAEF-YOLO consistently achieved superior precision–recall trade-offs, demonstrating enhanced detection accuracy and greater robustness in multi-task recognition.

**Figure 12:**
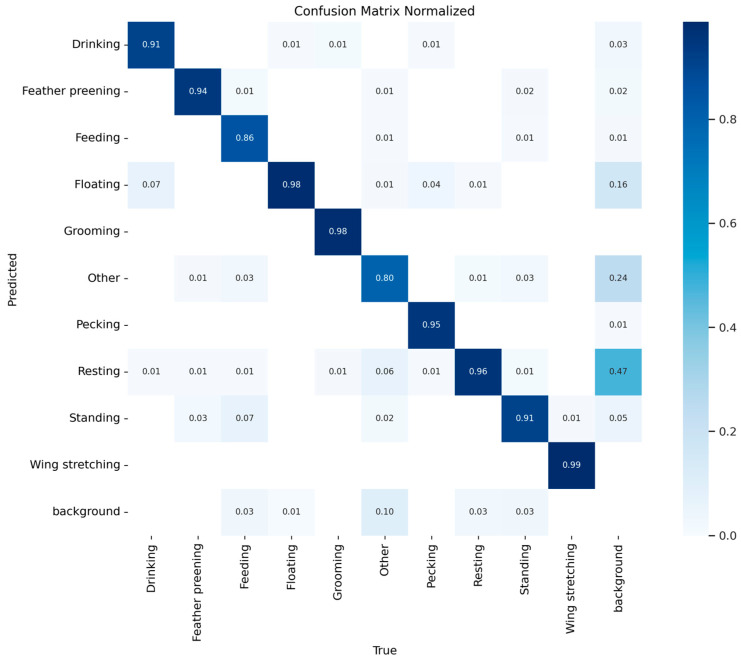
Normalized confusion matrix of the DAEF-YOLO model on the test set, showing per-class predictions versus ground truth labels for ten *Sanhua goose* behaviors and background.

Figure 12 presents the normalized confusion matrix of the DAEF-YOLO model on the test set. The model maintained consistently high true positive rates across most categories (e.g., Wing Stretching: 0.99; Grooming: 0.98; Floating: 0.98), confirming its robustness in fine-grained behavior recognition. However, several characteristic error patterns were observed. First, inter-class confusion primarily occurred between visually similar behaviors: Floating was occasionally misclassified as Resting (4%), and Feather Preening was sometimes mistaken for Grooming (3%). These misclassifications stem from subtle postural similarities under complex environmental conditions. Second, false positives appeared in background regions—most notably in Resting (47%) and Other (24%)—where water reflections or overlapping goose bodies were occasionally identified as behavioral instances.

Overall, DAEF-YOLO achieved excellent precision and recall across most categories; nonetheless, background interference and fine-grained behavioral similarity remain challenging. These findings highlight potential avenues for future improvement—such as incorporating temporal sequence modeling or multi-modal cues (e.g., depth or infrared imaging)—to further reduce misclassification under complex farming conditions.

In addition to the PR curves (Figure 11) and confusion matrix (Figure 12), Table 8 provides the quantitative per-class performance of the DAEF-YOLO model. It reports Precision, Recall, AP@0.5, and AP@0.5:0.95 for all ten behavioral categories. Majority classes—such as Floating and Resting—achieved consistently high metrics, whereas minority categories (e.g., Grooming, Wing Stretching) showed relatively lower AP values due to dataset imbalance, as discussed in Table 2. These findings confirm that the model maintains strong robustness for frequent behaviors, while also suggesting that targeted re-weighting or data augmentation strategies could further improve recognition of minority classes.

### 3.4. Comparative Experiments Between Different Models

To further evaluate the effectiveness of the proposed DAEF-YOLO model, comparative experiments were performed against several state-of-the-art object detection algorithms, including YOLOv5s, YOLOv7-Tiny, YOLOv7, YOLOv9s, and YOLOv10s [31]. The comparison focused on detection accuracy, model size, computational complexity, and inference speed.

As summarized in Table 9, all models were evaluated on the independent test set under identical training and inference conditions. Each model was executed three times, and since the variation across runs remained below 0.5%, only mean values are reported for clarity. The proposed DAEF-YOLO outperformed all baseline models, achieving Precision = 94.65%, Recall = 92.17%, F1 = 93.39%, mAP@0.5 = 96.10%, and mAP@0.5:0.95 = 69.82%.

**Figure 13:**
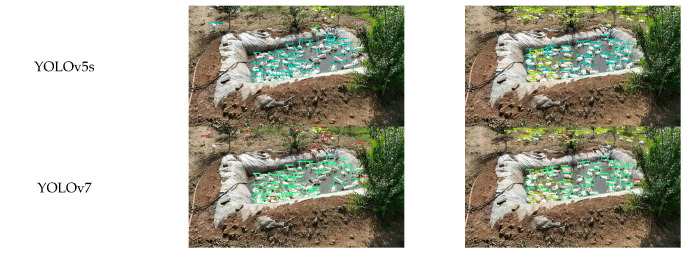

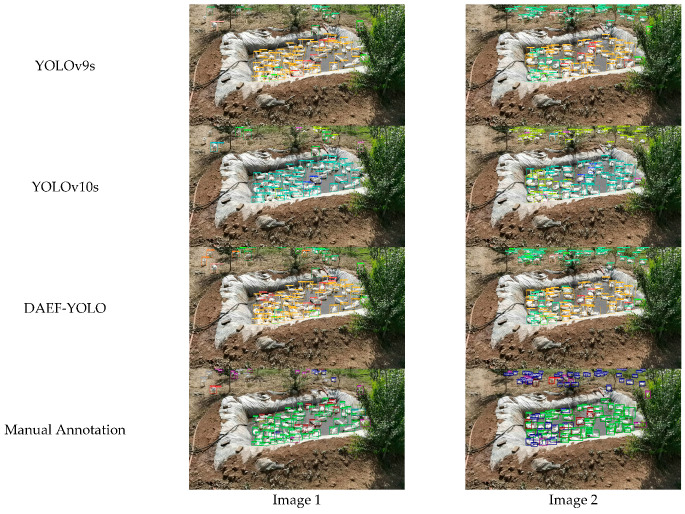
Image detection results of different object detection models on two representative test images.

To further verify the robustness of the improved model, four high-performing baselines (YOLOv5s, YOLOv7, YOLOv9s, and YOLOv10s) were compared with DAEF-YOLO on a representative subset of the test dataset. Detection confidence and IoU thresholds were uniformly set to 0.6 and 0.5, respectively. Representative detection outcomes are illustrated in Figure 13.

Additionally, Table 10 and Table 11 provide qualitative per-category analyses for two representative images: Image 1 (highest behavioral diversity) and Image 2 (highest individual count). These examples visually highlight inter-model performance differences, while the complete quantitative comparisons are summarized in Table 9. For Image 1, DAEF-YOLO detected 84 instances compared with 83 in manual annotation. For Image 2, the model perfectly matched the manual count of 117 targets, outperforming all other models in both total detection and per-category accuracy.

Overall, these results strongly confirm the superiority of DAEF-YOLO in dense, multi-class recognition of *Sanhua goose* behaviors. The model achieved simultaneously high recall and precision, even under occluded or cluttered conditions, demonstrating its robustness and strong potential for deployment in complex, real-world farming environments.

### 3.5. Heat Map Visualization Analysis

To further evaluate the feature detection capability of the proposed DAEF-YOLO model, Grad-CAM (Gradient-weighted Class Activation Mapping) heatmaps were generated. Specifically, Grad-CAM was computed from the C2f-Dual output at layer 12 of the YOLOv8 head, which provides a fused feature representation prior to the final detection stage. This configuration enables visualization of salient image regions that directly contribute to behavioral classification.

**Figure 14:**
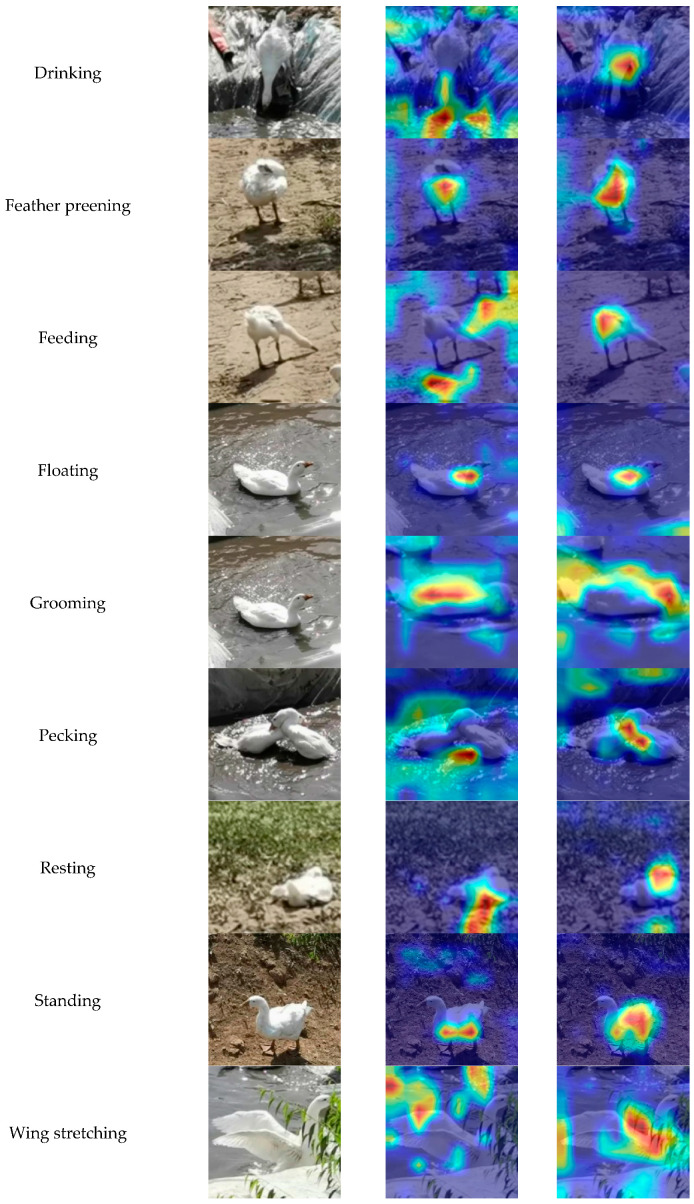

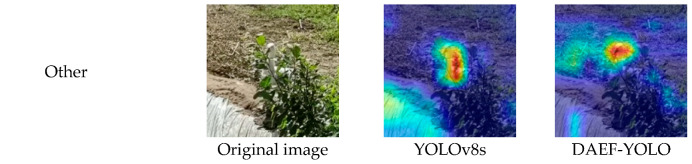
Comparison of Grad-CAM heatmaps for ten behavior categories before and after model optimization. Heatmaps were generated from the layer 12 (C2f-Dual) feature map of the YOLOv8 head. A consistent color bar was applied, with blue indicating low activation and red indicating high activation.

For interpretability, all Grad-CAM heatmaps were rendered using a consistent color bar across behavior categories, with blue representing low activation and red representing high activation. Figure 14 displays the Grad-CAM visualizations of YOLOv8s and DAEF-YOLO across representative behavioral classes.

Marked visual differences were observed between the two models. In behaviors such as Drinking, Feeding, Pecking, Resting, and Wing Stretching, YOLOv8s produced dispersed or background-biased activations, whereas DAEF-YOLO focused more precisely on the critical body regions of the geese. For Feather Preening, Grooming, Other, and Standing, DAEF-YOLO demonstrated stronger attention to discriminative areas such as the neck and wings, thereby improving classification accuracy. For instance, in Feather Preening, DAEF-YOLO emphasized the neck region—crucial for identifying this behavior—while YOLOv8s largely neglected it. Similarly, during Grooming, DAEF-YOLO accurately captured neck dynamics and water interaction cues, whereas YOLOv8s mainly concentrated on the torso region. In the “Other” category, DAEF-YOLO exhibited greater robustness to occlusion, maintaining attention even on partially visible heads.

Overall, the Grad-CAM analysis demonstrates that DAEF-YOLO achieves enhanced generalization and stronger activation responsiveness when detecting key behavioral features, significantly outperforming YOLOv8s in fine-grained behavior recognition tasks.

### 3.6. Ablation Study on the Effect of the “Other” Class

To evaluate the impact of introducing the “Other” category as a complementary class to the nine semantically defined behaviors, a controlled ablation study was performed under identical experimental settings (see Section 2.3). Two taxonomies were compared: (i) a 10-class system including “Other,” and (ii) a 9-class system excluding it. All ablation experiments were conducted on the test set, with each configuration independently repeated three times. Since the variation across runs consistently remained below 0.5%, only mean values are reported in Table 12 for clarity. The overall results are summarized below.

For YOLOv8s, excluding the “Other” class led to improvements in both mAP@0.5 (from 91.51% to 94.36%) and mAP@0.5:0.95 (from 69.91% to 73.68%), as expected when the number of classes decreases and decision boundaries become simpler. For DAEF-YOLO, mAP@0.5 remained nearly unchanged (96.10% vs. 96.08%), whereas mAP@0.5:0.95 was higher in the 9-class configuration (75.68% vs. 69.82%). This suggests that the 9-class taxonomy yields slightly higher strict AP values but sacrifices the ability to represent ambiguous or previously unmodeled behaviors.

Importantly, inclusion of the “Other” class ensures annotation completeness and provides a safety margin for ambiguous or infrequent postures that cannot be cleanly assigned to predefined categories. Although its per-class AP is relatively lower, this complementary class prevents label noise from contaminating well-defined categories, thereby improving model robustness in real-world farming conditions where unexpected or transitional postures frequently occur. Therefore, we retain the 10-class taxonomy including “Other” as the default configuration for practical deployment, while acknowledging that the 9-class variant may achieve marginally higher strict AP under simplified experimental conditions.

### 3.7. Multi-Task Capability: Individual Recognition Performance Under Different Annotation Strategies

To further assess the robustness of the DAEF-YOLO model in multi-task scenarios, we evaluated its individual recognition performance under two annotation strategies: (i) multi-class labeling, in which ten behavioral categories were preserved, and (ii) single-class labeling, in which all categories were merged into a single “goose” class while maintaining bounding-box positions. All experiments were conducted on the test set, and each configuration was independently run three times. Since the variation across runs consistently remained below 0.5%, only mean values are reported in Table 13 for clarity.

**Figure 15:**
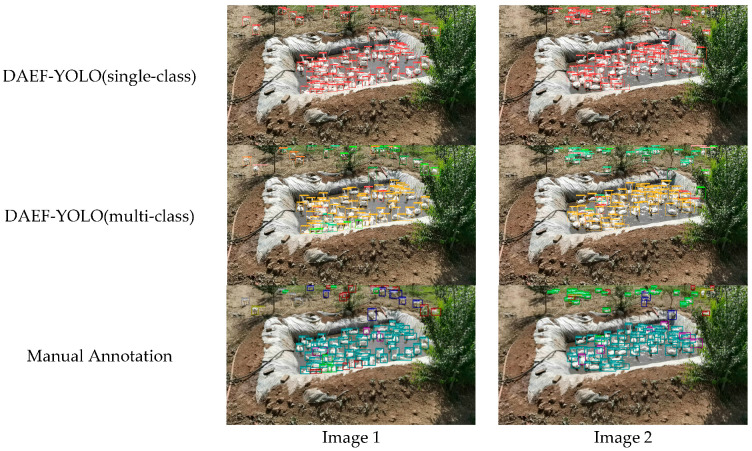
Representative qualitative examples of DAEF-YOLO detection results under multi-class and single-class labeling approaches. The model detected 86 geese (multi-class) vs. 88 geese (single-class) compared to 89 manually annotated in Image 1, and 105 (multi-class) vs. 101 (single-class) compared to 105 manually annotated in Image 2.

A comparative experiment was conducted on the same test dataset, evaluating both DAEF-YOLO and YOLOv8s under the two labeling configurations. Representative qualitative results are shown in Figure 15, and quantitative performance metrics are summarized in Table 13.

The results show that DAEF-YOLO maintained excellent performance under both single-class and multi-class settings, with particularly notable improvements over YOLOv8s in the more challenging multi-class detection task. Importantly, DAEF-YOLO achieved detection accuracy in multi-class labeling that was comparable to its single-class performance, indicating strong adaptability to complex recognition tasks.

To further validate whether the multi-class labeling strategy impaired individual-level counting, we conducted McNemar’s tests on the full test set (N = 344 images). For DAEF-YOLO, the contingency table yielded *a* = 117, *b* = 29, *c* = 22, *d* = 176, with χ^2^ = 0.961, *p* = 0.327. For YOLOv8s, the table yielded *a* = 93, *b* = 13, *c* = 6, *d* = 232, with an exact binomial McNemar’s test *p* = 0.167. McNemar’s test was applied following standard recommendations: chi-square approximation was used when the number of discordant pairs (*b* + *c*) ≥ 25, otherwise the exact binomial test was applied. In both models, *p* > 0.05, indicating no statistically significant difference between single-class and multi-class strategies. These results confirm that the proposed multi-class labeling approach does not degrade individual-level recognition performance (Table 14).

In summary, these findings demonstrate that DAEF-YOLO preserves efficient individual recognition capability while simultaneously distinguishing multiple behaviors. This confirms the model’s practical value in precision farming, as it successfully achieves recognition of ten distinct *Sanhua goose* behaviors without compromising individual-level detection accuracy.

## 4. Discussion

The effectiveness of livestock and poultry behavior recognition largely depends on the quality and diversity of the underlying datasets. However, existing goose behavior datasets remain limited in scale and diversity, primarily due to the challenges of continuous data collection, the high cost of manual annotation, and the broad variability of behavioral patterns. In this study, we conducted a statistical analysis of recent goose individual- and behavior-recognition research, including methods, behavior categories, and model performance, as summarized in Table 15. The results reveal that conventional datasets concentrate mainly on frequent behaviors such as standing, lying, and feeding, while rarely incorporating less common yet equally meaningful actions. Recognizing a broader behavioral spectrum is essential for continuous health monitoring and early detection of potential welfare or disease issues.

Compared with binary classification, multi-class frameworks can more effectively capture inter-behavior relationships and consequently improve recognition accuracy. Previous studies have shown that multi-label classification leverages co-occurrence relationships among categories while avoiding the information loss inherent in independent binary schemes [46]. Moreover, high-dimensional multi-class classification has been reported to strengthen weak feature learning and enhance model stability [47]. If a binary classification scheme had been used in this work, several behavioral categories would have been merged, thereby reducing their monitoring value. For example, feather preening and grooming are both self-maintenance behaviors but occur in distinct contexts; merging them would compromise the model’s discriminative capability. Thus, adopting a multi-class approach not only improves accuracy but also enables more granular behavioral analysis to support precision livestock management. Nevertheless, most current models still focus primarily on common goose behaviors, which limits their capacity to detect rare or abnormal patterns. This highlights the need to construct more comprehensive datasets that encompass a wider spectrum of behaviors, including those indicative of abnormalities. In this context, our study extends conventional categories by incorporating feather preening, floating, grooming, pecking, resting, standing, wing stretching, and an “Other” class, thereby enhancing both the diversity and practical value of *Sanhua goose* behavior recognition.

The 9-class taxonomy unsurprisingly achieves a higher mAP@0.5:0.95 because the classifier must distinguish among fewer decision boundaries. However, real farm footage often includes out-of-taxonomy or ambiguous postures. Without a complementary class, such instances are forcibly assigned to the nearest label, increasing inter-class confusion and compromising downstream decision logic. Introducing the “Other” class preserves annotation completeness, mitigates label noise, and enhances the multi-task detector’s reliability across different farms and seasons. Accordingly, we retain the 10-class taxonomy as the default configuration for deployment, whereas the 9-class variant may be preferable only when maximizing strict AP is the primary objective.

Although skeleton-based and GRU-driven frameworks [34,35] have achieved high accuracy in human- and driver-behavior recognition, their dependence on specialized sequential or skeleton annotations limits their applicability in livestock-farming contexts. In contrast, the proposed DAEF-YOLO processes raw RGB frames using only bounding-box annotations and achieves comparable detection accuracy, while demonstrating potential for deployment on embedded devices such as the Jetson Nano. Future work will further evaluate inference latency and resource utilization on such platforms. As summarized in Table 16, compared with representative state-of-the-art models beyond the YOLO family, DAEF-YOLO offers a favorable balance among accuracy, practicality, and scalability, underscoring its suitability for real-world goose-farming applications.

These results indicate that although skeleton-based and sequence-driven methods can achieve excellent accuracy in controlled environments, they encounter substantial barriers to adoption in real-world livestock farming. In contrast, DAEF-YOLO attains a well-balanced compromise between accuracy, practicality, and deployability, making it particularly suitable for precision-farming applications.

For example, Chuang et al. developed a visible- and infrared-thermal imaging system for monitoring goose surface temperature, achieving 97.1% accuracy in individual detection and regional segmentation using Mask R-CNN [4]. Li et al. proposed SDSCNet, a lightweight instance-segmentation network based on a depthwise-separable convolution encoder–decoder, which maintained high accuracy while reducing computational cost, enabling real-time deployment on devices such as the Raspberry Pi 4 [27]. Similarly, Xiao et al. designed DH-YOLOX with a dual-head structure and attention mechanism, allowing efficient recognition of key behaviors (e.g., drinking, resting, and standing) in group-raised Magang geese [5]. These studies collectively illustrate the evolution of deep-learning-based goose-behavior recognition—from individual detection to multi-class behavioral classification and health assessment—accompanied by a clear trend from reliance on high-performance computing platforms toward embedded-edge deployment, thereby advancing the practical realization of precision farming in the goose industry.

Despite these promising advances, several limitations persist. First, most existing studies still focus on single-task scenarios, constraining their ability to handle the complex requirements of detecting multiple individuals and behaviors in high-density farming environments. Second, many experiments are conducted under relatively controlled conditions, and therefore generalization to real-world settings—where lighting, occlusion, and background clutter vary substantially—remains inadequate.

To address these challenges, this study introduces the improved DAEF-YOLO model, explicitly designed for high-density and dynamically complex *Sanhua goose* farming environments. Our model enables simultaneous and efficient recognition of ten behaviors and individuals. In future work, we plan to extend this framework into an embedded intelligent system integrating automatic counting and density estimation, thereby advancing toward deployable technical solutions for precision livestock farming.

## 5. Conclusions

This study proposed an improved DAEF-YOLO model designed to address the challenges of recognizing ten distinct *Sanhua goose* behaviors under complex farming conditions. Additionally, a multi-task recognition strategy was introduced to enable simultaneous detection of individual geese and multiple behaviors, achieving individual-level accuracy comparable to single-category detection while effectively managing the complexity of multi-behavior scenarios. This strategy demonstrates strong adaptability and scalability, making it applicable to multi-task detection across various species, environments, and operational objectives.

Several architectural innovations contributed to the enhanced performance of the proposed model: the C2f-Dual module for multi-scale feature extraction, integration of ECA within the SPPF module to improve channel interaction efficiency, adoption of the FocalerIoU loss for more accurate bounding-box regression, and introduction of the ADown module for improved information retention during down-sampling. These enhancements collectively boosted accuracy while maintaining an optimal balance between computational cost and model size. Experimental results showed that DAEF-YOLO consistently outperformed mainstream object-detection models (YOLOv5s, YOLOv7-Tiny, YOLOv7, YOLOv9s, and YOLOv10s) across multiple evaluation metrics. Compared with YOLOv8s, DAEF-YOLO achieved improvements of 4.56%, 6.37%, 5.50%, and 4.59% in precision, recall, F1-score, and mAP@0.5, respectively—reaching 94.65%, 92.17%, 93.39%, and 96.10%. Validation results further confirmed that the model maintains robust individual-recognition capability while simultaneously identifying ten distinct behaviors.

Despite these advances, certain challenges remain—particularly in mitigating severe occlusion within high-density farming environments and optimizing deployment on mobile or low-power devices. Future research will focus on model optimization and compression to further enhance adaptability and real-world applicability. Integrating temporal modeling techniques (e.g., GRU or 3D skeleton-aware reasoning) into image-based detectors may further improve recognition of fast or subtle behaviors. Nonetheless, under current farming constraints, the proposed DAEF-YOLO provides an efficient and pragmatic solution that bridges the gap between advanced computer-vision models and the operational requirements of modern goose farming. Although real-time inference has been verified on server GPUs, further optimization and evaluation on embedded platforms such as Jetson Nano will be required to ensure reliable and practical deployability.

## Figures and Tables

**Table 1:** Behavior recognition rules for *Sanhua geese*.

Class	Definition	Instance
Drinking	Geese immerse their beaks in water to drink.	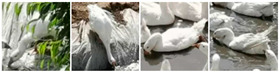
Feather preening	Geese contact their bodies with their beaks, often with their necks curved.	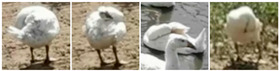
Feeding	Geese lower their heads to forage for food.	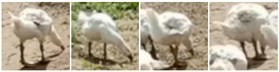
Floating	Geese float in water with their bodies relaxed.	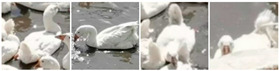
Grooming	Geese immerse their necks in water and move them back and forth to clean themselves.	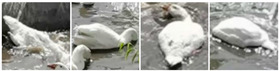
Pecking	One goose pecks at another goose with its beak.	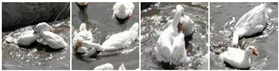
Resting	Geese lie on the ground or float on water, with their necks resting on their backs.	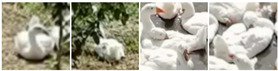
Standing	Geese maintain a standing posture or are walking.	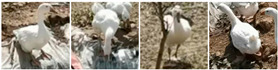
Wing stretching	Geese spread their wings, either to maintain balance or stretch their muscles.	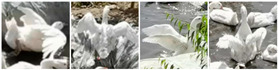
Other	Postures that cannot be clearly classified into the above nine behaviors, serving as their complement.	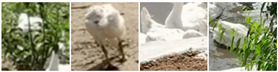

**Table 2:** The behavior instances distribution of the training, validation, and testing sets.

Class	Train_Set	Val_Set	Test_Set	Total
Drinking	2701	902	953	4556
Feather preening	1360	445	479	2284
Feeding	1018	313	293	1624
Floating	24,893	8571	8500	42,044
Grooming	673	226	229	1128
Pecking	1172	396	392	1960
Resting	24,478	8355	8715	41,548
Standing	2535	866	875	4276
Wing stretching	570	162	184	916
Other	5682	1888	1982	9552
All	65,082	22,124	22,682	109,888

**Table 3:** YOLOv8 model performance comparison. All training settings were identical to those described in Section 3.1 (Experimental Platform), and the reported FPS values were measured on the same GPU platform under this unified configuration.

Model	Depth	Width	mAP0.5 (%)	Model Size (MB)	Parameters	FPS (Frame/s)
YOLOv8n	0.33	0.25	86.01	6.4	3,012,798	97.9
YOLOv8s	0.33	0.50	91.51	22.7	11,139,454	74.3
YOLOv8m	0.67	0.50	95.12	52.2	25,862,110	28.6
YOLOv8l	1.00	1.00	96.04	87.8	43,637,550	26.9
YOLOv8x	1.00	1.25	96.33	136.0	68,162,238	22.2

**Table 4:** Comparison of detection accuracy and computational complexity between vanilla C2f and C2f-Dual in YOLOv8s.

Module Variant	P (%)	R (%)	F1 (%)	mAP0.5 (%)	Model Size (MB)	FLOPs (G)	Notes
Vanilla C2f (YOLOv8s)	90.09	85.80	87.89	91.51	22.7	28.7	Baseline
C2f-Dual (Position 1)	90.91	85.76	88.26	92.04	24.8	31.5	Replace C2f in backbone
C2f-Dual (Position 2)	91.23	86.23	88.66	92.40	25.2	31.5	Replace C2f in neck
C2f-Dual (Pos. 1 + 2)	91.61	87.11	89.30	92.94	27.4	34.3	Replace both

Note: Model sizes were obtained from exported FP32 weights; FLOPs were computed under the same input resolution as in the experiments.

**Table 5:** Effect of different loss functions on model detection performance.

Loss Function	Precision (%)	Recall (%)	F1 (%)	mAP@0.5 (%)
CIoU (YOLOv8s)	90.09	85.80	87.89	91.51
DIoU	91.71	85.42	88.45	92.10
SIoU	91.46	85.92	88.60	92.35
GIoU	91.00	86.29	88.58	92.42
FocalerIoU	91.54	87.13	89.28	93.26

**Table 6:** Hardware and Hyperparameter Information. Training time per epoch and throughput are reported for the DAEF-YOLO model.

Configuration Item	Value
Input image size	1280 × 1280 × 3 pixels
CPU	AMD EPYC 7542, 32-core processor
GPU	2 × NVIDIA GeForce RTX 4090 (24 GB memory per GPU)
RAM	128 GB
Operating system	Ubuntu 20.04.6
Programming language	Python 3.11
Framework	PyTorch 2.2.1
CUDA Version	12.1.1
Optimizer	Stochastic Gradient Descent (SGD)
Initial learning rate	0.01
Momentum	0.937
Weight decay	0.0005
Batch size	8
Epochs	100
LR schedule	Step schedule with linear warmup (3 ep)
EMA	Not used
Early stopping	Not used (patience = 100)
Mixed precision	Disabled (FP32 training)
Training time/epoch	1.42 min (DAEF-YOLO)
Throughput	9.73 img/s (DAEF-YOLO)

**Table 7:** Ablation test results of different model configurations.

Model	C2f-Dual	SPPF-ECA	FocalerIoU	ADown	P (%)	R (%)	F1 (%)	mAP@0.5 (%)	mAP@0.5:0.95 (%)	Model Size (MB)	FLOPs (G)	FPS (Frame/s)
YOLOv8s	×	×	×	×	90.09	85.80	87.89	91.51	69.91	22.7	28.7	74.3
A	√	×	×	×	91.61	87.11	89.30	92.94	71.16	27.4	34.3	74.9
B	×	√	×	×	92.26	87.17	89.64	92.95	71.03	27.9	30.8	87.7
C	×	×	√	×	91.54	87.13	89.28	93.26	71.32	22.7	28.7	89.2
D	√	√	×	×	92.35	86.62	89.39	92.95	71.40	32.6	36.5	69.9
E	√	×	√	×	90.73	88.96	89.84	93.80	71.79	27.4	34.4	78.7
F	×	√	√	×	92.86	87.83	90.27	93.62	71.33	27.9	38.8	86.2
G	×	√	√	√	93.50	89.24	91.32	94.39	72.68	25.7	28.0	67.5
H	√	√	√	√	94.65	92.17	93.39	96.10	71.50	30.4	33.8	82.9

**Table 8:** Per-class Precision, Recall, AP@0.5, and AP@0.5:0.95 for *Sanhua goose* behavior recognition on the test set (DAEF-YOLO, 10-class taxonomy).

Class	P (%)	R (%)	AP@0.5 (%)	AP@0.5:0.95 (%)
Drinking	84.99	88.53	96.12	73.03
Feather preening	88.59	84.89	97.68	74.32
Feeding	85.83	84.67	94.73	66.41
Floating	97.38	93.49	99.08	78.08
Grooming	95.14	94.7	99.34	79.03
Other	82.98	85.26	83.69	46.16
Pecking	91.83	93.14	97.74	74.75
Resting	96.85	93.84	97.47	66.51
Standing	90.26	86.81	95.72	65.26
Wing stretching	95.99	96.44	99.38	76.35
Overall (mean)	94.65	92.17	96.10	69.82

**Table 9:** Comparison results of different network models for goose behavior recognition. All models were trained on the same *Sanhua goose* dataset under identical training settings, and the results were evaluated on the independent test set to ensure a fair comparison.

Model	P (%)	R (%)	F1 (%)	mAP@0.5 (%)	mAP@0.5:0.95 (%)	Model Size (MB)	FLOPs (G)	FPS (Frame/s)
YOLOv5s	72.31	66.05	69.04	73.68	50.00	15.1	16.0	78.6
YOLOv7-Tiny	76.17	69.63	72.75	77.21	48.47	12.6	13.3	144.9
YOLOv7	91.27	84.58	87.80	91.61	65.14	75.1	103.3	68.4
YOLOv8s	90.09	85.80	87.89	91.51	69.91	22.7	28.7	74.3
YOLOv9s	87.18	82.34	84.69	88.90	66.96	19.5	39.6	74.6
YOLOv10s	84.00	80.46	82.19	87.23	65.80	16.7	24.8	111.1
DAEF-YOLO	94.65	92.17	93.39	96.10	69.82	30.4	33.8	82.9

**Table 10:** Comparison of detection results of different models on Image 1 (test set example).

Class	YOLOv5s	YOLOv7	YOLOv9s	YOLOv10s	DAEF-YOLO	Manual Annotation
All	57	74	70	61	84	83
Drinking	0	7	5	2	8	9
Feather preening	1	1	1	1	1	1
Feeding	1	0	0	0	5	1
Floating	48	48	49	46	48	46
Grooming	0	0	0	0	0	1
Pecking	0	0	0	0	0	1
Resting	4	5	5	5	5	5
Standing	1	8	6	5	10	8
Wing stretching	0	0	0	0	0	0
Other	2	5	4	2	7	11

**Table 11:** Comparison of detection results of different models on Image 2 (test set example).

Class	YOLOv5s	YOLOv7	YOLOv9s	YOLOv10s	DAEF-YOLO	Manual Annotation
All	93	107	101	94	117	117
Drinking	0	5	5	3	5	5
Feather preening	0	1	1	1	1	1
Feeding	0	1	0	0	0	1
Floating	41	40	40	40	44	41
Grooming	0	0	0	0	0	0
Pecking	0	0	0	0	0	0
Resting	51	56	52	46	60	60
Standing	0	0	0	0	1	0
Wing stretching	0	0	0	0	0	0
Other	1	4	3	4	6	9

**Table 12:** Ablation on the “Other” class (overall metrics). YOLOv8s and DAEF-YOLO trained under identical pipelines with either a 10-class taxonomy (with “Other”) or a 9-class taxonomy (without “Other”).

Model	Classes	P (%)	R (%)	F1 (%)	mAP@0.5 (%)	mAP@0.5:0.95 (%)
YOLOv8s	9 (without Other)	92.61	88.84	90.65	94.36	73.68
YOLOv8s	10 (with Other)	90.09	85.80	87.86	91.51	69.91
DAEF-YOLO	9 (without Other)	94.50	91.00	92.75	96.08	75.68
DAEF-YOLO	10 (with Other)	94.65	92.17	93.34	96.10	69.82

**Table 13:** Performance evaluation of DAEF-YOLO versus YOLOv8s across annotation strategies.

Model	P (%)	R (%)	F1 (%)	mAP@0.5 (%)	mAP@0.5:0.95 (%)
YOLOv8s(single-class)	97.20	94.82	96.00	97.82	75.26
YOLOv8s(multi-class)	90.09	85.80	87.89	91.51	69.91
DAEF-YOLO(single-class)	97.89	96.00	96.94	98.57	73.87
DAEF-YOLO(multi-class)	94.65	92.17	93.39	96.10	71.50

**Table 14:** Image-level McNemar’s test comparing single-class vs. multi-class labeling strategies on the full test set (N = 344 images).

Model	Item	Value
DAEF-YOLO	a (both correct)	117
	b (1-only correct)	29
	c (10-only correct)	22
	d (both wrong)	176
	b + c	51
	Method	Chi-square approximation
	χ^2^ statistic	0.961
	*p*-value	0.327
	Accuracy (1-class)	0.424
	Accuracy (10-class)	0.404
	Conclusion	Not significant (*p* > 0.05)
YOLOv8s	a (both correct)	93
	b (1-only correct)	13
	c (10-only correct)	6
	d (both wrong)	232
	b + c	19
	Method	Exact binomial McNemar test
	χ^2^/Exact statistic	–
	*p*-value	0.167
	Accuracy (1-class)	0.308
	Accuracy (10-class)	0.288
	Conclusion	Not significant (*p* > 0.05)

**Table 15:** Comparison of the proposed DAEF-YOLO with representative non-YOLO state-of-the-art behavior recognition models.

Model	Input Modality	Application Domain	Reported Performance	Notes on Applicability to Farming Scenarios
GRU-based skeleton dynamic graph [34]	Skeleton sequences	Human gesture recognition	Accuracy ≈ 94%	Requires skeleton joint data; not feasible for large-scale goose flocks
3D skeleton-aware driver behavior recognition [35]	3D skeleton + temporal data	Driver monitoring	Accuracy ≈ 95%	Relies on motion capture or skeleton extraction; limited transferability
DAEF-YOLO (this study)	RGB images	Goose individual and behavior recognition	P = 94.65%, R = 92.17%, mAP@0.5 = 96.10%	Operates directly on raw farm video; deployable on embedded devices

**Table 16:** Recent Statistics of Behavior Categories in Goose Individual and Behavior Recognition Studies.

Livestock	Methods	Categories	Performance(%)			
			mAP	Accuracy	Precision	Recall
White Roman goose [4]	Mask R-CNN (Instance Segmentation)+ Visible camera + Infrared thermal camera integration on Jetson Xavier NX	Single class: goose (individual detection for surface temperature monitoring)			97.1	95.1
Sichuan white goose [27]	SDSCNet—instance segmentation network with depthwise separable convolution encoder–decoder, INT8 quantization for embedded deployment	Single class: goose (instance segmentation of ~80 individuals, no behavior categories)		93.3		
Magang goose [5]	DH-YOLOX—improved YOLOX with dual-head structure and attention mechanism for key behavior detection	Multi-class: drinking, foraging, other non-feeding and dinking, cluster rest, cluster stress	98.98			

## Data Availability

The datasets generated, used, and/or analyzed during the current study will be available from the corresponding author upon reasonable request.

## Supplementary Materials

The following supporting information can be downloaded at: https://www.mdpi.com/article/10.3390/ani15203058/s1, Table S1. Data augmentation operations with trigger probabilities, parameter ranges, bounding-box handling, and notes.
