# Supplementary material for: DAEF-YOLO Model for Individual and Behavior Recognition of Sanhua Geese in Precision Farming Applications
Source: Animals (Basel). 2025 Oct 21;15(20):3058. doi: 10.3390/ani15203058 (PMC12561539; doi:10.3390/ani15203058)
Supplement: Supplementary file 1 [file animals-15-03058-s001.zip › animals-3863750-supplementary.pdf]

Supplementary Materials

Table S1. Data augmentation operations with trigger probabilities, parameter ranges, bounding-box handling, and notes.

Operation	Trigger probability	Parameter range / setting	Bounding box handling	Notes
Rotation	≈0.50	Angle ∈ [−5°, +5°]; Scale ∈ [0.70, 0.80]	Re-projected by affine transform; bounding box retightened by min. enclosing rectangle	Random.uniform; enforced at least one augmentation
Translation	≈0.50	Horizontal shift ∈ [−(d_left−1)/3, +(d_right−1)/3]; Vertical shift ∈ [−(d_top−1)/3, +(d_bottom−1)/3]	Shifted synchronously	Ensured no object left image
Brightness	≈0.50	$\alpha \in [0.35, 1.00]$; output = $\alpha \cdot I + (1-\alpha)\cdot 0$	N/A	Implemented via OpenCV addWeighted
Gaussian noise	≈0.50	Mode=gaussian; $\sigma^2=0.01$ (in [0,1] space)	N/A	skimage.random_noise, output rescaled to 0–255
Cutout	≈0.50	1 hole; side length=50 px; IoU≤0.50 with any GT box	If IoU>0.5, resample location	Prevents masking more than 50% of any object
Flipping	≈0.50	Vertical / horizontal / both; ≈1/3 each	Bboxes mirrored accordingly	Implemented via cv2.flip
Mosaic/Mixup	–	Not used	–	Avoided to reduce distributional shift
Random seed	–	random.seed(2024), numpy.random.seed(2024)	Global seed	Same across baselines for fair comparison
